# Symptom Network and Lesion Mapping of Post‐Stroke Depression, Cognitive Impairment, and Dysfunction: A Multicenter Cohort Study

**DOI:** 10.1002/brb3.71759

**Published:** 2026-09-10

**Authors:** Qing Du, Wenwen Liang, Yifan Fang, Tianyi Li, Chensheng Pan, Guo Li, Zhou Zhu

**Affiliations:** ^1^ Department of Neurology, Tongji Hospital, Tongji Medical College Huazhong University of Science and Technology Wuhan Hubei China

**Keywords:** ischemic stroke, network analysis, voxel‐based lesion–symptom mapping

## Abstract

**Background:**

This study aimed to examine the interplay among depressive symptoms, cognitive impairment, and dysfunction after ischemic stroke using a network analysis approach, and to investigate whether selected network‐derived targets show convergent neuroanatomical correlates using voxel‐based lesion–symptom mapping (VLSM) in an imaging subset.

**Methods:**

We prospectively enrolled 589 acute ischemic stroke patients and constructed a 3‐month post‐stroke symptom network. Depression, cognition, and dysfunction were assessed via 17‐item Hamilton Depression Rating Scale (HDRS), Mini‐Mental State Examination (MMSE), and modified Rankin Scale (mRS), respectively. Flow analysis quantified dysfunction's direct association with neuropsychiatric symptoms. In an imaging subset (*n* = 429), VLSM localized neuroanatomical substrates of key network targets.

**Results:**

*Depressed mood *and* Psychiatric anxiety *were central symptoms, while *Suicidality, Loss of interest, Psychiatric anxiety*, and* mRS score* acted as bridge symptoms*. mRS score* was positively associated with* Suicidality and Loss of interest*, and negatively associated with *Language*. VLSM identified a significant cluster in the left superior corona radiata for* Language*, whereas* Suicidality *and* Loss of interest* showed no focal anatomical correlates.

**Conclusions:**

Identifying core and bridge symptoms (e.g., *Depressed mood*, *Psychiatric anxiety*, *Suicidality*, *Loss of interest*, *mRS score*) may serve as candidate indicators for rehabilitation planning. *Suicidality*, *Loss of interest* and *Language* may serve as promising candidates for monitoring and rehabilitation. Notably, while *Language* maps to the left superior corona radiata, the absence of focal lesions for *Suicidality* and *Loss of interest* suggests reliance on distributed network dysfunction.

## Graphical abstract

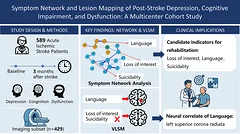

## 1 Introduction

Stroke remains one of the leading causes of long‐term disability and reduced quality of life worldwide (Feigin et al. 2018). The first 3 months following a stroke represent a critical period for neuroplasticity (T. A. Jones and Adkins 2015). Early rehabilitation, once the patient is medically stable and safe to mobilize, helps maximize functional recovery. Post‐stroke depression (PSD) and post‐stroke cognitive impairment (PSCI) rank among the most frequent neuropsychiatric complications following a stroke. PSD can delay recovery and increase mortality, whereas PSCI often impairs independence and social participation (Kapoor et al. 2019; Li et al. 2022; Ribeiro and Madruga 2021). Consequently, early, symptom‐specific psychological and rehabilitative interventions are essential for optimizing long‐term recovery.

The mechanisms linking post‑stroke depression, cognitive impairment, and functional disability remain poorly understood, which hinders the development of integrated rehabilitation strategies that address these comorbidities together. To map the conditional dependencies within these multifaceted symptom clusters, network analysis provides a robust empirical framework. Diverging from conventional univariate or aggregated scoring systems, this methodology isolates direct structural relationships and pinpoints central hubs along with bridging elements across diverse clinical domains. In stroke survivors, prior network evaluations have designated symptoms such as fatigue, pessimism regarding the future, and extremity pain as pivotal bridging or core components (Huang et al. 2024). Parallel investigations demonstrated that emotional nodes, including sadness and guilt, alongside diminished verbal output, serve as structural links connecting depressive and functional impairments (Lau et al. 2022). Furthermore, attentional and psychomotor deficits frequently mediate the interaction between affective and cognitive networks (Shi et al. 2024). Ultimately, while these scattered insights reveal distinct domain overlaps, they highlight the need for integrative analytical approaches to better understand the clustering of post‑stroke symptoms.

Beyond the interplay among symptoms, where a lesion occurs also shapes neuropsychiatric and cognitive outcomes after stroke. Damage involving the left superior corona radiata has been associated with worse global disability and ambulatory outcomes, and voxel‐wise mapping implicates the corona radiata in domain‐specific functional deficits (e.g., swallowing) (Kim et al. 2021; Moulton et al. 2015; Wilmskoetter et al. 2019). Voxel‐based lesion–symptom mapping (VLSM) studies further show that brain–behavior relations often track specific symptom components rather than broad syndromes (Bates et al. 2003). However, our measures are relatively coarse, and VLSM results based on these measures should therefore be considered exploratory. Accordingly, in addition to constructing the post‐stroke symptom network in our cohort, we applied VLSM in the imaging subset to explore whether network‐derived targets show distinct neuroanatomical correlates. We did not have a strong prior hypothesis for any specific region, given the multifactorial nature of post‑stroke mood and cognitive symptoms.

## 2 Materials and Methods

### 2.1 Study Design

This multicenter prospective cohort study included a cross‐sectional network analysis at 3 months to support early screening and prevention of post‐stroke complications. Local ethics approval was secured for the protocol. All participants consented in writing, consistent with the Declaration of Helsinki.

### 2.2 Participants

A total of 687 patients were recruited from three tertiary hospitals in Wuhan, China (Tongji Hospital, Wuhan First Hospital, and Wuhan Central Hospital) between May 2018 and August 2019. The study duration for each patient was from admission to the 3‐month follow‐up visit. Consecutive adults (≥ 18 years) with imaging‐confirmed ischemic stroke admitted to three tertiary hospitals within 7 days of onset were screened. We excluded non‐vascular brain disease, pre‐stroke psychiatric disorder or dementia, severe aphasia or cognitive impairment precluding assessment, transient ischemic attack, subarachnoid hemorrhage, other major neurological disorders, and inability to complete follow‐up. Clinical and demographic data were taken from records and bedside examination. In total, 589 patients completed the 3‐month visit; magnetic resonance imaging (MRI) suitable for lesion mapping was available in 429 patients. The inclusion flow is shown in Supporting Information Figure S1.

### 2.3 Measurement

Selection bias was minimized through consecutive recruitment, and measurement bias was reduced by standardized training assessments.

#### 2.3.1 Depressive Symptom Assessment

To evaluate depressive symptoms, we used the 17‑item Hamilton Depression Rating Scale (HDRS)—a tool that many clinicians and researchers consider the reference standard for gauging depression severity (Boessen et al. 2013; Meader et al. 2014). Of the 17 HDRS items, we excluded item 14 (*Genital symptoms*) because 578 of 589 patients (98.1%) scored 0. Item 17 (*Insight*) was excluded because it showed very low variance (527 of 589 patients, 89.5%, scored 0). We did not rely on a fixed statistical cut‐off alone, as node selection in psychological network approaches should combine quantitative variance with clinical and theoretical relevance (Borsboom et al. 2021). Item 17 was excluded not only because of low variance but also because its clinical meaning in stroke patients is ambiguous: Insight depends heavily on cognitive and emotional factors, so including it would likely add measurement noise (Bagby et al. 2004). Item 3 (*Suicidality*) was retained because about 10% of patients scored ≥ 1 (mean = 0.14, SD = 0.46; full distribution in Supporting Information Table S4). A sensitivity analysis excluding this item confirmed that it did not meaningfully affect the network structure (edge‑weight correlation = 0.990; see Results). Items 4, 5, and 6 (*Initial*, *Nocturnal*, *and Late insomnia*, respectively) overlapped topologically, and we also computed Cronbach's *α* for the three insomnia items using our cohort data, which was 0.77, indicating acceptable internal consistency. It exceeds the conventional threshold of 0.70, supporting the aggregation of these items into a single composite score. That left 13 items for the network analysis. Of note, the mean score for *Suicidality* was 0.14 (range 0–4), and scores of 3 or higher, which would indicate active suicidal ideation, were extremely rare. Thus, in our cohort, this item primarily captured passive thoughts about life not being worth living rather than active suicidal ideation.

#### 2.3.2 Cognitive Assessment

The Mini‐Mental State Examination (MMSE) measured cognitive performance across four domains (orientation, memory, attention, language) and served as an established screening tool for cognitive impairment (Truong et al. 2024). Total scores range from 0 to 30, and the MMSE has been extensively applied in research on postoperative and post‐stroke cognitive dysfunction. We scored the MMSE according to five standard subdomains: Orientation (10 items), Registration (three items), Attention (five items), Memory (three items), and Language (nine items). Each subdomain total was entered as a separate node in the network.

#### 2.3.3 Functional Impairment Assessment

The modified Rankin Scale (mRS), developed from the original 1957 Rankin Scale and refined in 1988, is now the standard measure of post‐stroke dysfunction (New and Buchbinder 2006; Sulter et al. 1999; van Swieten et al. 1988). It evaluates functional independence across body function, activity, and participation, using a six‐point scale from 0 (*no symptoms*) to 5 (*severe disability*; Sulter et al. 1999).

### 2.4 Network Estimation

The present investigation utilized the R packages qgraph (Epskamp et al. 2012) and bootnet (Epskamp, Borsboom, and Fried 2018). The network, which comprised depressive symptoms, cognitive symptoms, and the global functional outcome (*mRS score*), was estimated using a Gaussian graphical model (GGM)—an undirected network model (Epskamp, Waldorp, et al. 2018). Similar to previous studies (Y. Zhang et al. 2024), we estimated the network using Spearman correlations—because items are ordinal, data may be skewed, and low‑frequency cells can distort polychoric correlations. Each node stood for a depressive, cognitive, or the functional outcome (*mRS score*). Edges reflected partial correlations adjusted for every other node. For regularization, we used both the least absolute shrinkage and selection operator (LASSO) and the extended Bayesian information criterion (EBIC). The integration of these methodologies collectively facilitated the contraction of all edge weights and forced negligible correlations to absolute zero (Epskamp, Borsboom, and Fried 2018). The hyperparameter for the EBIC was defined as 0.1, a relatively liberal value that favors sensitivity over specificity, as our aim was exploratory, and we preferred to include potentially meaningful connections that might be relevant for future hypothesis generation. Edge thickness reflected the strength and sign of associations (blue = positive; red = negative). Predictability for each node, estimated via mgm, quantified the variance explained by neighboring nodes and was visualized as circular indicators (Haslbeck and Waldorp 2020). Finally, the flow function in qgraph was employed to visualize direct and indirect associations between cognitive and depressive items and dysfunction.

To examine whether the network structure differed by depression severity, we additionally performed a subgroup analysis using the NetworkComparisonTest (NCT). The NCT compares network structures between two independent groups by testing differences in global strength and overall network structure through permutation testing. We compared patients with HDRS ≥ 8 (*n* = 229) to an equal‑sized randomly selected subsample of patients with HDRS < 8 (*n* = 229), using 1000 permutation iterations. The significance level was set at *p* < 0.05.

#### 2.4.1 Sensitivity Analysis Excluding the Dysfunction Node

The mRS score is a global disability measure, not a symptom. To see whether its presence in the network altered connections among the 18 symptom nodes, we re‑estimated the network without mRS (same estimation settings). We then compared edge weights (Spearman correlation, median and 95th percentile absolute differences), expected influence (EI), and bridge EI (BEI) between the original 18‑node subgraph and the new 18‑node network. Strong agreement would imply that the core symptom structure is independent of including the mRS node.

### 2.5 Centrality and Bridging Centrality Estimation

EI and BEI were computed using qgraph and networktools to identify central and bridging nodes (Epskamp et al. 2012; P. J. Jones et al. 2021). EI was chosen for its superior performance in mixed‐edge networks (McNally 2021; Robinaugh et al. 2016). EI equaled the sum of incident edge weights without absolute values (Robinaugh et al. 2016). In contrast, node strength aggregates absolute magnitudes. Using signed weights lets EI determine if a node exerts an excitatory or inhibitory role on the network (Robinaugh et al. 2016).

To locate the most impactful nodes in relation to cognitive impairment, depression, and dysfunction, the Bridging BEI index was considered appropriate (Burger et al. 2023; P. J. Jones et al. 2021). Nodes with BEI values above the 80th percentile were classified as bridge nodes (P. J. Jones et al. 2021), indicating high potential to contribute to comorbidity and symptom transmission between communities. Additional bridging indices are reported in the Supporting Information. Centrality measures were reported as normalized values (“Z‐scores”).

### 2.6 Network Stability and Accuracy

Each network model underwent accuracy and stability evaluation via the bootnet package. Cases with missing data were excluded. We performed 1000 case‐dropping bootstraps to obtain correlation stability (CS) coefficients for EI and BEI, with CS coefficients ≥ 0.25 considered acceptable and > 0.5 preferable (Epskamp, Borsboom, and Fried 2018). Edge‐weight accuracy was evaluated using 1000 nonparametric bootstraps to derive 95% confidence intervals (CIs), with narrower CIs indicating more reliable estimates. Centrality difference tests and edge‐weight difference tests were used to assess whether nodes and edges differed significantly within each network.

### 2.7 Age Comparison of Network Characteristics

This study utilized a cutoff age of 60 years to examine potential outcome differences between age groups. In accordance with prior research (Zheng et al. 2025), we used the NCT from the R package to examine age‐related differences in network characteristics. Edge‐weight distributions were compared across networks to assess structural variation, and group differences in edge strength were tested after Holm–Bonferroni correction for multiple comparisons.

### 2.8 Image Acquisition and Preprocessing

Baseline clinical MRI scans were obtained from 429 participants. An experienced rater blind to behavioral data manually traced lesions on diffusion‐weighted imaging (DWI) using ITK‐SNAP version 3.8.0 (www.itksnap.org). A second neurologist then reviewed all segmentations for consistency. Spatial normalization of MRI and lesion masks to MNI152 was done with the Clinical Toolbox (Pan et al. 2022) in SPM12 (Wellcome Trust Centre for Neuroimaging, London, UK) running on MATLAB R2021a (The MathWorks, Natick, MA). Normalized lesion maps were visually checked and manually adjusted if needed. Lesion volumes were obtained from the normalized segmentations in ITK‑SNAP. Finally, all lesion maps were overlaid on a template in MRIcron 1.0 (Chris Rorden, Columbia, SC; www.nitrc.org/projects/mricron) to illustrate the spatial distribution of lesions in the study cohort.

### 2.9 VLSM

To test whether lesion location relates to flow‑network symptom targets, we performed VLSM in NiiStat (Stark et al. 2017). Each target was binarized using clinical cutoffs (*Language* ≤ 6; *Loss of interest*≥2; *Suicidality*≥ 1). Dichotomization is common in VLSM when symptoms reflect categorical impairment or rare events (Bates et al. 2003). One‑tailed Liebermeister tests were run on voxels lesioned in ≥ 5 patients (Weaver et al. 2021). To correct for multiple comparisons, we used voxel‐level family‐wise error (FWE) with 10,000 random permutations. We set the threshold for results at P(FWE) below 0.05 on the voxel level. Significant clusters were localized in MRIcron: the Automated Anatomical Labeling 3 (AAL3) atlas (Rolls et al. 2020) and the JHU‐WhiteMatter‐labels‐1 mm atlas.

### 2.10 Clinical Rationale for Dichotomizing Network‐Derived Targets

In neuropsychiatric assessments, *Suicidality* and *Loss of interest* often have many zeros and a strong right skew. Standard linear models assume normality and equal variance—violated here—which can bias standard errors, inflate false positives, and distort effect sizes (Ren et al. 2024). To avoid this, we binarized these variables for VLSM using prespecified cutoffs. Converting ordinal scales into binary categories separates impairment from intact function, trading subclinical variance for diagnostic validity—a standard neuropsychiatric approach (Bates et al. 2003; Kimberg et al. 2007). *Language* cutoff (1 SD below the cohort mean) follows common practice for domain impairment (Jak et al. 2009). *Loss of interest* and *Suicidality* were coded as present versus absent based on any clinically relevant endorsement, consistent with depression scale usage (Zimmerman et al. 2013) and with epidemiologic/clinical standards for *Suicidality* (Posner et al. 2011).

## 3 Results

### 3.1 Sample Characteristics

The demographic profiles of the study population were summarized in Supporting Information Table S1. The mean MMSE was 26.16 (SD = 3.60) and the mean HDRS was 6.93 (SD = 5.97), indicating an overall mild symptom burden, with 39% (*n* = 229) of patients having HDRS ≥ 8. The names, means, standard deviations, EIs and BEIs of nodes in the depression‐cognition‐dysfunction network of the ischemic stroke patients are shown in Table 1. In the imaging subset (*n* = 429), the most frequently affected regions were the basal ganglia and thalamus (Supporting Information Figure S12).

**TABLE 1:** Mean, SD, EI, and BEI for each symptom in the depression‐cognition‐dysfunction network (n  =  589).

Node name	Symptoms	Mean (SD)	EI	BEI
D1	Depressed mood	0.80 (1.03)	2.18	0.41
D2	Feelings of guilt	0.40 (0.59)	0.83	0.45
D3	Suicidality	0.14 (0.46)	0.66	0.91
D4	Insomnia	1.44 (1.73)	0.038	0.12
D5	Loss of interest	0.51 (0.92)	0.73	1.35
D6	Retardation	0.29 (0.53)	−0.79	−1.12
D7	Agitation	0.47 (0.68)	−0.19	0.02
D8	Psychiatric anxiety	0.90 (1.02)	1.78	1.03
D9	Somatic anxiety	0.46 (0.73)	0.45	0.59
D10	Gastrointestinal somatic	0.28 (0.50)	−0.43	−0.76
D11	General somatic	0.35 (0.61)	0.26	0.35
D12	Hypochondriasis	0.36 (0.67)	0.48	0.59
D13	Weight loss	0.36 (0.70)	−0.80	0.18
C1	Orientation	9.50 (1.10)	−0.84	−1.32
C2	Registration	2.87 (0.45)	−0.37	0.64
C3	Attention	3.94 (1.45)	−0.18	−0.59
C4	Memory	2.32 (0.85)	−0.84	−1.35
C5	Language	7.52 (1.40)	−1.93	−2.45
DF	mRS score	1.15 (1.00)	−1.06	0.95

### 3.2 Network Structure

**FIGURE 1:**
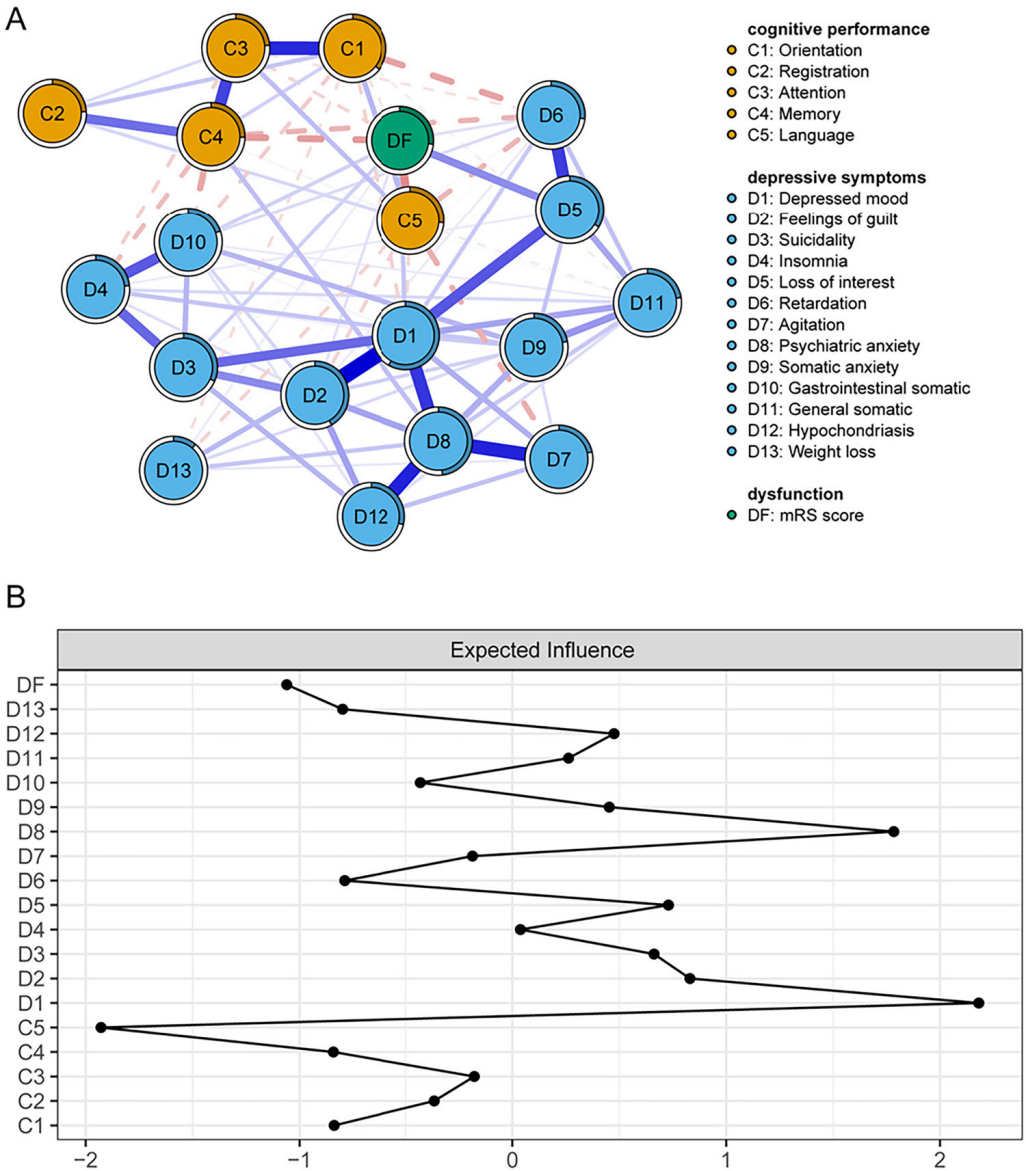
Network structure and node centrality.  *Note*: Blue edges indicate positive relations, with thicker and more saturated edges denoting stronger connections between symptom nodes. Nodes with stronger connections are closer to each other.

The depression–cognition–dysfunction network in ischemic stroke patients is shown in Figure 1A, and a correlation matrix is shown in Supporting Information Table S2. The network consists of 19 nodes. In the depressive symptom subnetwork, D1 (*Depressed mood*)–D2 (*Feelings of guilt*), D1 (*Depressed mood*)–D8 (*Psychiatric anxiety*), D5 (*Loss of interest*)–D6 (*Retardation*), D7 (*Agitation*)–D8, and D8–D12 (*Hypochondriasis*) were strongly associated. In the cognitive performance subnetwork, C1 (*Orientation*)–C3 (*Attention*) and C3–C4 (*Memory*) were strongly associated.

### 3.3 Central Nodes and Bridge Nodes

In Figure 1B, D1 has the highest centrality, followed by D8 and D2, whose EI values are significantly higher than those of most other nodes.

**FIGURE 2:**
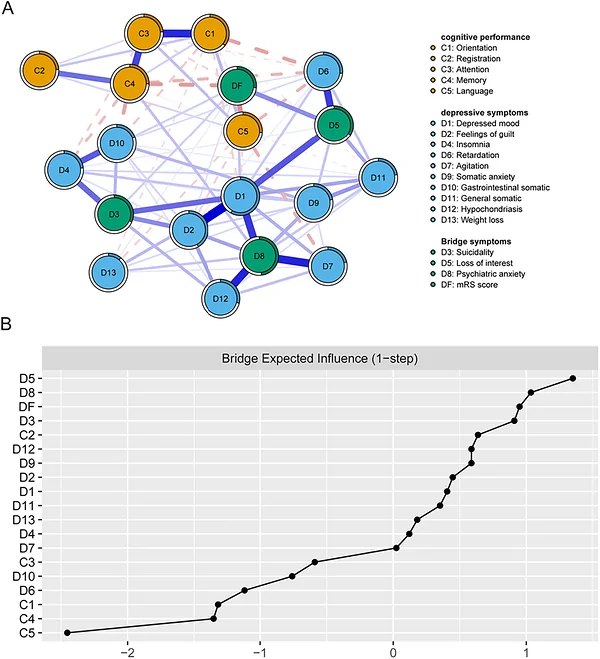
Bridge symptoms across communities. *Note*: Blue edges represent positive relations, with thicker and more saturated edges denoting stronger connections between symptom nodes. Nodes with stronger connections are closer to each other.

Figure 2A shows the bridge nodes. In Figure 2B, D3 (*Suicidality*), D5, D8, and DF (*mRS score*) had the highest BEIs in their communities and acted as bridge nodes. Results were unchanged after excluding extreme edge weights (no covariate adjustment).

### 3.4 Network Stability and Accuracy

**FIGURE 3:**
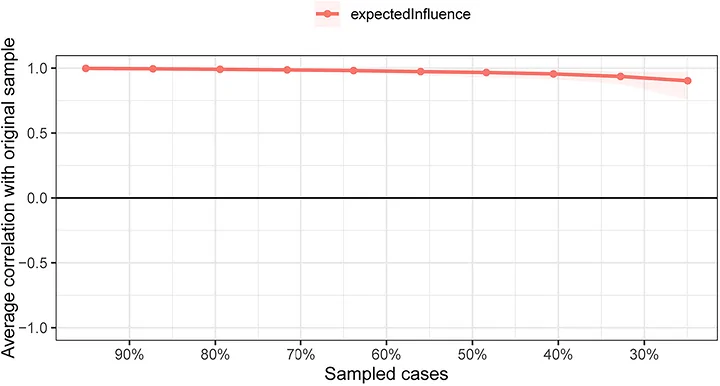
Stability of node expected influences in the network. *Note*: The red bar represents the average correlation between node expected influences in the full sample and subsample with the red area depicting the 2.5th to the 97.5th quantile.

**FIGURE 4:**
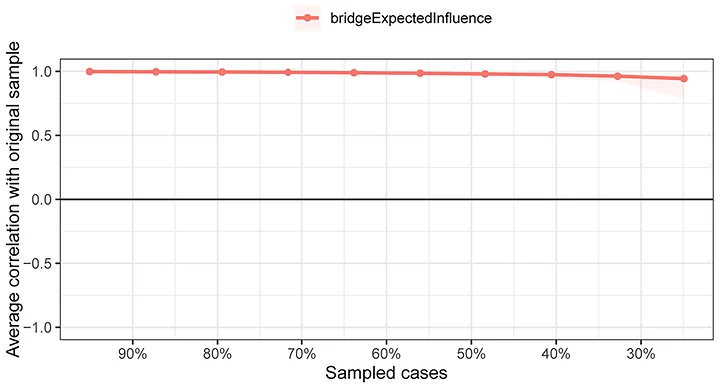
Stability of node bridge expected influences in the network. *Note*: The red bar represents the average correlation between node bridge expected influences in the full sample and subsample with the red area depicting the 2.5th to the 97.5th quantile.

EI and BEI both had CS > 0.5 (Figures 3 and 4). Bootstrap difference tests showed that EI values for D1, D2, and D8 were significantly higher than for other nodes (Supporting Information Figure S6). For BEI, three bridging nodes (D3, D5, D8) had significantly higher values than other nodes (Supporting Information Figure S7).

Supporting Information Figure S4 shows a narrow 95% CI from the bootstrap, meaning edge weight estimates were accurate and stable. Supporting Information Figure S5 reports bootstrap difference test results: Seven edges had significantly higher weights than the rest.

Network stability metrics beyond node and bridge centrality are shown in Supporting Information Figures S8 and S9.

### 3.5 Flow Network of Dysfunction

**FIGURE 5:**
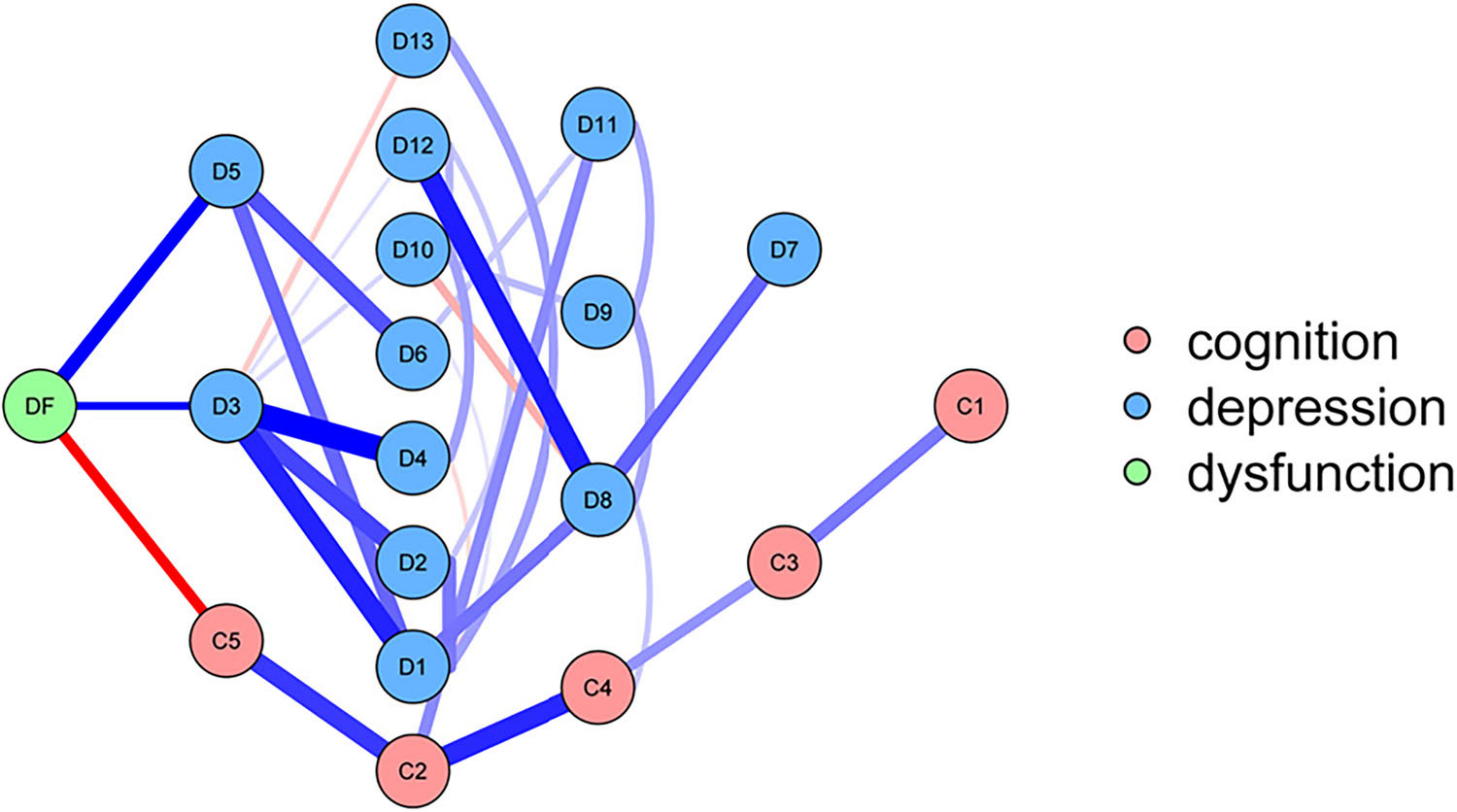
Flow network of DF. *Note*: The nodes in the network represent depressive symptoms, cognition items, and dysfunction in the post‐stroke patients, and the edges represent correlations of symptoms. Blue edges and red edges represent positive and negative correlations, respectively, and thicker edges represent higher correlations. And the meanings of nodes: D1 = Depressed mood, D2 = Feelings of guilt, D3 = Suicidality, D4 = Insomnia, D5 = Loss of interest, D6 = Retardation, D7 = Agitation, D8 = Psychiatric anxiety, D9 = Somatic anxiety, D10 = Gastrointestinal somatic, D11 = General somatic, D12 = Hypochondriasis, D13 = Weight loss, C1 = Orientation, C2 = Registration, C3 = Attention, C4 = Memory, C5 = Language, DF = mRS score.

There were three nodes directly associated with DF and 15 symptoms indirectly associated with DF (Figure 5). Among the direct edges of DF, the strongest positive edge was D5 (edge weight = 0.25), followed by D3 (edge weight = 0.15), and the strongest negative edge was C5 (edge weight = −0.19).

### 3.6 Age Effects

There were no significant age‐group differences in network strength (6.63 vs. 5.28, *p* = 0.73) or edge weights (M = 0.21, *p* = 0.28; Supporting Information Figure S10).

### 3.7 VLSM

**FIGURE 6:**
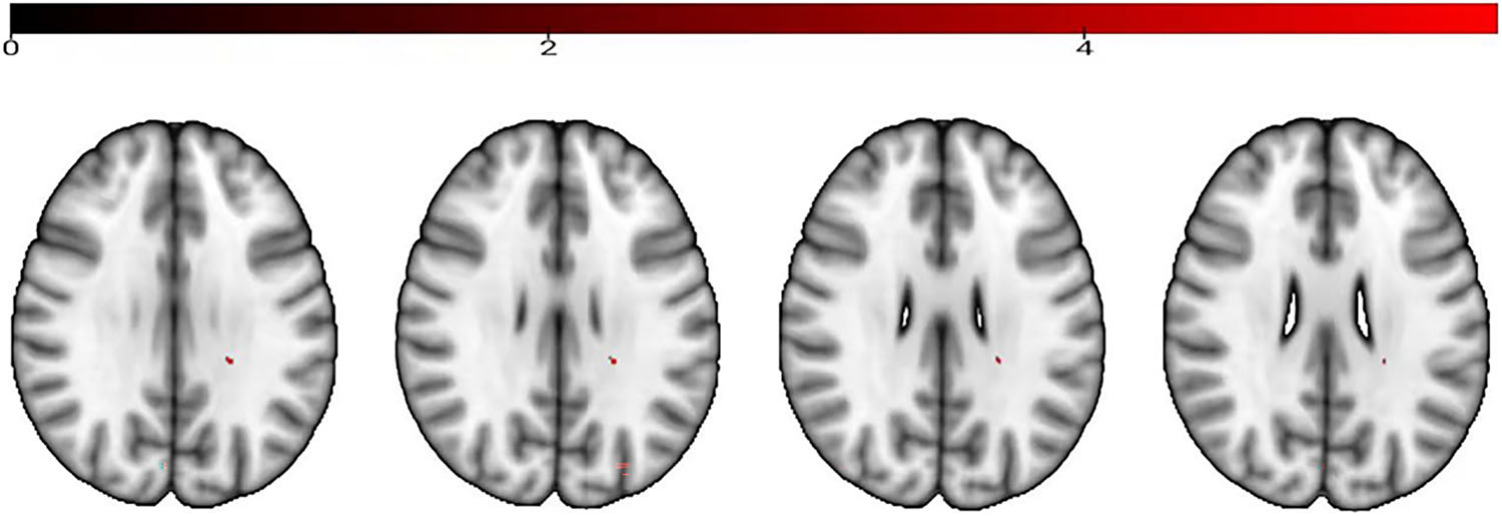
VLSM results for binary *Language*. Voxels shown indicate regions where lesions are significantly more frequent in patients with language impairment than in those without. The significant cluster is in the left superior corona radiata.

Of three flow‑network targets, VLSM linked only *Language* to left superior corona radiata lesions (35 voxels, FWE‑corrected). *Loss of interest* and *Suicidality* showed no clusters. The peak Montreal Neurological Institute (MNI) coordinate of the significant cluster (35 voxels) for *Language* was [−26, 4, 28] (Table 2, Figure 6).

**TABLE 2:** Detailed statistics for target VLSM analyses

Target	Maximum Z value	FWE‐corrected threshold	N of significant voxels	Peak MNI (x,y,z)	Location of significant voxels
Language	4.989775	z > 4.61537	35	−26, 4, 28	left superior corona radiata
Loss of interest	4.892871	z > 4.61537	5	—	Unclassified^a^
Suicidality	3.986817	z > 4.68534	0	—	—

*Note*: FWE family‐wise error. Positive Z values indicate voxels where lesions are more frequent in patients with target scores at or above the cut‐off than in those below the cut‐off.

Unclassified^a^ indicates that the significant voxels do not belong to any gray matter region or white matter tract in AAL and “JHU‐WhiteMatter‐labels‐1 mm” atlases.

Abbreviation: MNI: Montreal Neurological Institute.

### 3.8 Sensitivity Analysis: Exclusion of the Dysfunction Node

The two 18‑node networks (with vs. without *mRS score*) showed nearly identical edges (*ρ* = 0.993) and perfect EI ranking (*ρ* = 1.00). Core/bridge nodes were largely unchanged. Thus, *mRS score* does not meaningfully alter the symptom architecture (Supporting Information Table S3). This indicates that *mRS score* is peripheral to the symptom network and does not materially alter the connectivity among depressive and cognitive symptoms.

### 3.9 Sensitivity Analysis for Depression Severity

The global strength was 3.152 in the HDRS ≥ 8 group and 2.322 in the HDRS < 8 group. The observed difference was not significant (S = 0.830, *p* = 0.308). Similarly, the overall network structure did not differ between groups (M = 0.203, *p* = 0.771). These results indicate that the connectivity pattern is preserved across depression severity levels and is not merely an artefact of contrasting symptomatic versus asymptomatic patients.

## 4 Discussion

We applied network modeling to characterize how depressive, cognitive, and functional symptoms interact after stroke, and combined these analyses with flow‐network visualization and VLSM. This multimodal approach highlighted a small set of depressive and cognitive symptoms as potential markers for monitoring and candidate nodes for future rehabilitation research at the 3‑month post‑stroke stage.

Within the psychological network, *Depressed mood*, *Feelings of guilt*, and *Psychiatric anxiety* showed the highest centrality, with *Depressed mood* emerging as the most influential node. Strong connections among depressed mood and guilt and anxiety are consistent with cognitive models of depression, which emphasize tightly coupled negative self‐appraisal and anxious preoccupation (Obonsawin et al. 2017). More links between anxiety and other signs of depression (e.g., D7–D8 and D8–D12) show that *Psychiatric anxiety* is a central factor that may maintain and spread emotional problems. Clinically, these findings indicate that rehabilitation planning focusing on *Depressed mood*, *Feelings of guilt*, and *Psychiatric anxiety*—via pharmacotherapy, cognitive restructuring, or emotion‐focused psychotherapy—may have an outsized association with the overall post‐stroke symptom network, encompassing subsequent cognitive and functional outcomes.

Bridge analyses further identified *mRS score*, *Suicidality*, *Loss of interest*, and *Psychiatric anxiety* as key connectors between psychological symptoms and global disability. In our cohort, however, *Suicidality* exhibited a low baseline severity (mean score = 0.14). Its role as a bridge symptom should therefore be understood as a marker of emotional distress that may be related to recovery perceptions, not as an indicator of acute suicide risk. Accordingly, we do not suggest this finding supports suicide risk screening but rather that even passive low‑level thoughts may warrant clinical attention as indicators of broader emotional distress. Prior work has shown that psychological distress and illness perceptions are closely related in stroke survivors and are also associated with mental health and recovery trajectory (Pai et al. 2019), and network studies in other mental‐health samples highlight *Feelings of guilt and Loss of interest* as central drivers of broader psychopathology (N. Zhang et al. 2023). Our results extend these observations by showing that, after stroke, *Loss of interest* and *Suicidality* are positioned to be associated with mood disturbance and functional impairment. *Loss of interest* tends to become more prominent in later stages of stroke recovery and has been associated with fatigue and reduced life satisfaction (Hu et al. 2025), whereas severe medical illnesses such as myocardial infarction and stroke are known to increase the risk of suicidal behavior (Scott et al. 2010).

Flow‐network analyses showed that *Loss of interest* and *Suicidality* had the strongest positive links with dysfunction, consistent with prior reports linking anhedonia after stroke to poorer outcomes and less favorable rehabilitation (Pan et al. 2023; Segura et al. 2024). However, this association should be interpreted with caution, as the HDRS item for *Loss of interest* includes anchor descriptions that partially overlap with functional impairment measured by the mRS, which may have inflated the observed correlation. It should be noted, however, that *Suicidality* in our cohort primarily reflected passive thoughts about life not being worth living rather than active suicidal ideation. Beyond single symptoms, growing evidence suggests that emotion‐regulation difficulties are associated with symptom severity and that emotion‐regulation strategies may improve psychosocial outcomes in stroke survivors (Balram et al. 2025). Evidence from ecological assessments further established that self‐regulation and emotion‐regulation strategies can weaken the momentary coupling between depressive symptoms and activity participation (Lee et al. 2024). Our network findings, particularly regarding *Loss of interest* and *Suicidality*, are consistent with the hypothesis that emotion regulation strategies warrant further investigation as potential avenues for improving psychosocial outcomes in stroke survivors. However, direct evidence in post‐stroke cohorts remains limited, and future longitudinal and interventional studies are needed to examine whether interventions that address these symptoms are associated with improved functional outcomes.

Cognitive and language outcomes emerged as an additional axis along which symptom networks and functional recovery intersect. Language ability is a key marker of post‐stroke cognition, and interventions such as speech therapy and cognitive training can promote cognitive recovery and improve quality of life and independence (Ginex et al. 2017). Several studies have reported close coupling between cognitive recovery and global functional outcomes (Fast et al. 2023; Park et al. 2017). In line with this, *Language* in our network was closely related to dysfunction. These findings are consistent with multidisciplinary rehabilitation approaches in which psychotherapists, speech–language therapists, and physical therapists jointly address mood and cognitive–language deficits in relation to functional outcomes (Hu et al. 2025; Jaracz et al. 2002).

By integrating flow‐network analysis with VLSM, we further examined the neural substrates of post‐stroke symptoms. Two network‐central affective nodes—*Loss of interest* and *Suicidality*—did not show robust focal lesion correlates at the voxel level. This may suggest that these phenomena reflect distributed network disruption or diaschisis (Griffis et al. 2019). In contrast, *Language* localized to the left superior corona radiata, a major white‐matter conduit interconnecting perisylvian language cortices and long‐range association pathways such as the superior longitudinal fasciculus, which have repeatedly been linked to language performance and recovery after stroke (Kyeong et al. 2019; Schevenels et al. 2022). Clinically, the convergence between a network‐central symptom (*Language*) and a structural locus (left superior corona radiata) suggests that this symptom may be a promising candidate for monitoring in language rehabilitation and for future investigations of pathway‑level neuromodulation or task‑oriented training. Concurrently, neuroimaging investigations among clinical depression cohorts established that neural circuit dysfunction within reward processing systems potentially modulates suicidal ideation through the mediation of anhedonia (Wang et al. 2023), suggesting that similar distributed network mechanisms may also be relevant to post‐stroke mood disturbances. Future work using multivariate lesion–symptom mapping, structural disconnection measures, and connectome‐based approaches may help capture such symptoms more fully and delineate how structural disconnection and symptom networks jointly constrain recovery.

The language measure used was a single MMSE subitem, which is coarse and cannot capture fine‐grained distinctions between language subdomains (e.g., production vs. comprehension; Pendlebury et al. 2010). In addition, patients with severe aphasia were excluded from the study because they could not complete the psychometric assessments, which may have restricted the range of language deficits and reduced the power to detect more extensive lesion correlates (Shiggins et al. 2022). Thus, the identified cluster in the left superior corona radiata should be interpreted with caution and requires replication in larger, more severely affected cohorts with dedicated language batteries. The absence of focal correlates for *Suicidality* and *Loss of interest* is consistent with the view that these symptoms arise from distributed network dysfunction rather than from a single anatomical site (Bates et al. 2003). Indeed, bridge centrality describes a property of the symptom network's conditional dependence structure and does not imply that a bridging symptom necessarily has a distinct neuroanatomical substrate. Future studies using connectivity‐based or multivariate lesion‐symptom mapping approaches may be better suited to capture their neural substrates.

This study has several limitations. First, the cross‐sectional design prevents inference about temporal or causal relations between stroke severity, depressive symptoms, and cognition; longitudinal follow‐up would provide stronger evidence. Second, although tests for differences in edge weights and centrality are widely used in symptom network research (Campbell and Osborn 2021; Ross et al. 2020), current approaches to multiple‐comparison correction are imperfect, and improved methods are still needed (Epskamp, Borsboom, and Fried 2018). Third, recruitment from tertiary hospitals may have introduced selection bias and limited generalizability. Fourth, cognition was assessed only with the MMSE. Although the MMSE is widely used in stroke research and allows comparison with previous studies, it has limited sensitivity to executive and visuospatial functions and may show ceiling effects in patients with mild cognitive deficits after stroke (Pendlebury et al. 2010). This could have reduced the connectivity estimates among cognitive nodes and between cognitive and other domains, potentially underestimating the role of executive dysfunction in the overall network (Pendlebury et al. 2010). Fifth, our cohort consisted mostly of patients with mild stroke and relatively low symptom burden. This limits generalizability to more severely affected populations and may reduce the variability needed to detect certain associations. In addition, the mRS is a relatively coarse measure of functional outcome; in our mild cohort, most patients were clustered at the lower end of the scale (scores 1–2), which may have limited its discriminative ability to detect nuanced associations between functional status and neuropsychiatric symptoms. Beyond this, the HDRS includes somatic items that may be elevated due to stroke‑related physical symptoms, and the *Loss of interest* contains anchor descriptions that partially overlap with functional impairment measured by the mRS. These measurement‑related factors should be considered when interpreting associations between depressive symptoms and functional outcomes. Finally, the VLSM analysis was restricted to the imaging subset and was limited by the use of a coarse MMSE subitem for *Language* and the exclusion of patients with severe aphasia, which may have constrained the variability in language performance. Given the low base rates of *Loss of interest* and *Suicidality* and the limited sensitivity of univariate lesion–symptom mapping for distributed network effects, null results for these targets may partly reflect limited power. Furthermore, findings regarding the *Suicidality* node should be interpreted with caution, as this item had a very low mean score and primarily captured passive thoughts rather than active suicidal ideation. Future studies using connectivity‑based or multivariate approaches with more comprehensive assessments in diverse cohorts are warranted.

## 5 Conclusion

In this multicenter cohort of acute ischemic stroke, symptom‐network and flow analyses suggested that post‐stroke dysfunction is embedded in a broader pattern of depressive and cognitive symptoms rather than reflecting global severity alone. In the flow network, *Loss of interest* and *Suicidality* showed the strongest positive links with dysfunction, whereas better language performance was negatively related, suggesting that these features are key nodes in the depression–cognition–dysfunction system. VLSM tied *Language* to lesions in the left superior corona radiata but found no robust focal correlates for *Loss of interest* or *Suicidality*, highlighting these symptoms as clinically important markers for monitoring and multidisciplinary rehabilitation planning.

## Author Contributions

**Wenwen Liang**: Writing – original draft, software, Writing – review and editing. **Qing Du**: writing – original draft, conceptualization, methodology, writing – review and editing. **Chensheng Pan**: validation, formal analysis. **Tianyi Li**: data curation, validation. **Zhou Zhu**: supervision, funding acquisition, visualization, project administration, resources. **Guo Li**: writing – original draft, validation, formal analysis. **Yifan Fang**: data curation, investigation.

## Funding

This work was financially supported by the National Natural Science Foundation of China [Grant Number 82171465] and Wuhan Key Research and Development Program Project [Grant Number 2025020102030017]. The funders had no role in study design, data collection and analysis, decision to publish, or preparation of the manuscript.

## Ethics Statement

The approval of the study for experiments was obtained from the Ethics Committee of Tongji Medical College, Huazhong University of Science and Technology (TJ‐IRB20171108). The registration number of this prospective cohort study was ChiCTR‐ROC‐17013993. The URL of the publicly accessible website on which this trial is registered is: http://www.chictr.org.cn/index.aspx. All procedures were in accordance with the Declaration of Helsinki.

## Conflicts of Interest

The authors declare no conflicts of interest.

## Supporting information




**Supporting File 1**: brb371759‐sup‐0001‐SuppMat.docx

## Data Availability

The de‐identified database used in the current study is available from the corresponding author on reasonable request.
