# Supplementary material for: Symptom Network and Lesion Mapping of Post‐Stroke Depression, Cognitive Impairment, and Dysfunction: A Multicenter Cohort Study
Source: Brain Behav. 2026 Sep 10;16(9):e71759. doi: 10.1002/brb3.71759 (PMC13563064; doi:10.1002/brb3.71759)
Supplement: Supplementary file 1 [file BRB3-16-e71759-s001.docx]

**Supplementary material**

**Supplementary Table 1.** General characteristics of the analysis sample(n=589).

| Characteristic | Value |
| --- | --- |
| Gender, n (%) |  |
| Male | 458(78%) |
| Female | 131(22%) |
| Age, years, mean (SD), range | 59.28 (10.70), 22-86 |
| Education, n (%) |  |
| Education without schooling | 47(8.0%) |
| High school education | 138(23%) |
| Junior high school education | 187(32%) |
| Primary school education | 124(21%) |
| University education | 93(16%) |
| Marital status, n (%) |  |
| Divorced | 7(1.2%) |
| Married | 554(94%) |
| Unmarried | 8(1.4%) |
| Widowed | 20(3.4%) |
| Clinical scores |  |
| HDRS total score, mean (SD) | 6.93 (5.97) |
| HDRS ≥ 8, n (%) | 229(39%) |
| MMSE total score, mean (SD) | 26.16 (3.60) |
| mRS score, mean (SD) | 1.15 (1.00) |

Note: HDRS = 17-item Hamilton Depression Rating Scale; MMSE = Mini‑Mental State Examination; mRS = modified Rankin Scale.

Percentages may not sum to 100% due to rounding.

**Supplementary Table 2.** The edge weights within the depression-cognition-dysfunction network.

|  | C1 | C2 | C3 | C4 | C5 | D1 | D2 | D3 | D4 | D5 | D6 | D7 | D8 | D9 | D10 | D11 | D12 | D13 | DF |
| --- | --- | --- | --- | --- | --- | --- | --- | --- | --- | --- | --- | --- | --- | --- | --- | --- | --- | --- | --- |
| C1 | 0.000 |  |  |  |  |  |  |  |  |  |  |  |  |  |  |  |  |  |  |
| C2 | 0.062 | 0.000 |  |  |  |  |  |  |  |  |  |  |  |  |  |  |  |  |  |
| C3 | 0.249 | 0.040 | 0.000 |  |  |  |  |  |  |  |  |  |  |  |  |  |  |  |  |
| C4 | 0.050 | 0.167 | 0.218 | 0.000 |  |  |  |  |  |  |  |  |  |  |  |  |  |  |  |
| C5 | 0.082 | 0.044 | 0.071 | 0.013 | 0.000 |  |  |  |  |  |  |  |  |  |  |  |  |  |  |
| D1 | -0.050 | 0.000 | 0.000 | 0.000 | -0.044 | 0.000 |  |  |  |  |  |  |  |  |  |  |  |  |  |
| D2 | 0.000 | 0.000 | 0.000 | -0.006 | -0.049 | 0.296 | 0.000 |  |  |  |  |  |  |  |  |  |  |  |  |
| D3 | 0.000 | 0.000 | 0.000 | 0.000 | 0.000 | 0.172 | 0.135 | 0.000 |  |  |  |  |  |  |  |  |  |  |  |
| D4 | 0.000 | 0.000 | -0.051 | -0.073 | 0.000 | 0.018 | 0.035 | 0.174 | 0.000 |  |  |  |  |  |  |  |  |  |  |
| D5 | 0.000 | 0.000 | 0.000 | 0.000 | -0.011 | 0.195 | 0.000 | 0.000 | 0.023 | 0.000 |  |  |  |  |  |  |  |  |  |
| D6 | -0.105 | 0.000 | -0.033 | -0.084 | -0.082 | 0.060 | 0.022 | 0.009 | 0.000 | 0.226 | 0.000 |  |  |  |  |  |  |  |  |
| D7 | 0.000 | 0.000 | 0.000 | 0.000 | -0.089 | 0.089 | 0.000 | 0.000 | 0.000 | 0.000 | 0.000 | 0.000 |  |  |  |  |  |  |  |
| D8 | 0.000 | 0.000 | 0.000 | 0.071 | 0.000 | 0.233 | 0.105 | 0.000 | 0.000 | 0.000 | 0.000 | 0.255 | 0.000 |  |  |  |  |  |  |
| D9 | 0.000 | 0.000 | 0.000 | 0.000 | 0.000 | 0.060 | 0.051 | 0.004 | 0.064 | 0.066 | 0.000 | 0.039 | 0.092 | 0.000 |  |  |  |  |  |
| D10 | -0.056 | 0.000 | -0.057 | -0.100 | 0.000 | 0.013 | 0.047 | 0.084 | 0.191 | 0.000 | 0.039 | 0.000 | 0.000 | 0.082 | 0.000 |  |  |  |  |
| D11 | -0.022 | 0.008 | 0.000 | 0.000 | -0.023 | 0.103 | 0.000 | 0.000 | 0.043 | 0.101 | 0.069 | 0.000 | 0.062 | 0.119 | 0.023 | 0.000 |  |  |  |
| D12 | 0.000 | 0.000 | 0.000 | 0.000 | 0.000 | 0.000 | 0.108 | 0.082 | 0.000 | 0.000 | 0.000 | 0.070 | 0.257 | 0.055 | 0.032 | 0.037 | 0.000 |  |  |
| D13 | -0.037 | 0.000 | 0.000 | 0.000 | -0.048 | 0.079 | 0.000 | 0.000 | 0.000 | 0.006 | 0.006 | 0.026 | 0.064 | 0.000 | 0.000 | 0.041 | 0.000 | 0.000 |  |
| DF | -0.031 | 0.000 | -0.046 | -0.114 | -0.136 | 0.065 | 0.033 | 0.051 | 0.050 | 0.132 | 0.034 | 0.000 | 0.000 | 0.000 | 0.000 | 0.000 | 0.000 | 0.021 | 0.000 |

Note: D1 = Depressed mood, D2 = Feelings of guilt, D3 = Suicidality, D4 = Insomnia, D5 = Loss of interest, D6 = Retardation, D7 = Agitation, D8 = Psychiatric anxiety, D9 = Somatic anxiety, D10 = Gastrointestinal somatic, D11 = General somatic, D12 = Hypochondriasis, D13= Weight loss, C1 = Orientation, C2 = Registration, C3 = Attention, C4 = Memory, C5 = Language, DF= mRS score

**Supplementary Table 3.** Comparison of network centrality metrics between the 18‑symptom subgraph (derived from the original 19‑node network) and the re‑estimated 18‑node network (excluding mRS).

| Node | Symptom | EI (original subgraph) | EI (re‑estimated, no mRS) | ΔEI | BEI (original subgraph) | BEI (re‑estimated, no mRS) | ΔBEI |
| --- | --- | --- | --- | --- | --- | --- | --- |
| D1 | Depressed mood | 1.22 | 1.24 | 0.02 | -0.09 | -0.11 | -0.02 |
| D2 | Feelings of guilt | 0.74 | 0.73 | 0.01 | -0.55 | -0.75 | -0.2 |
| D3 | Suicidality | 0.66 | 0.68 | 0.02 | 0 | -0.01 | -0.01 |
| D4 | Insomnia | 0.42 | 0.43 | 0.01 | -0.12 | -0.14 | -0.02 |
| D5 | Loss of interest | 0.61 | 0.63 | 0.02 | -0.01 | -0.03 | -0.02 |
| D6 | Retardation | 0.13 | 0.13 | 0 | -0.30 | -0.33 | -0.03 |
| D7 | Agitation | 0.39 | 0.39 | 0 | -0.09 | -0.09 | 0 |
| D8 | Psychiatric anxiety | 1.14 | 1.15 | 0.01 | 0.07 | 0.08 | 0.01 |
| D9 | Somatic anxiety | 0.63 | 0.63 | 0 | 0 | -0.01 | -0.01 |
| D10 | Gastrointestinal somatic | 0.30 | 0.30 | 0 | -0.21 | -0.22 | -0.01 |
| D11 | General somatic | 0.56 | 0.57 | 0.01 | -0.04 | -0.04 | 0 |
| D12 | Hypochondriasis | 0.64 | 0.65 | 0.01 | 0 | 0 | 0 |
| D13 | Weight loss | 0.14 | 0.14 | 0 | -0.09 | -0.09 | 0 |
| C1 | Orientation | 0.17 | 0.17 | 0 | -0.27 | -0.29 | -0.02 |
| C2 | Registration | 0.32 | 0.34 | 0.02 | 0.01 | 0.01 | 0 |
| C3 | Attention | 0.44 | 0.45 | 0.01 | -0.14 | -0.15 | -0.01 |
| C4 | Memory | 0.25 | 0.25 | 0 | -0.19 | -0.23 | -0.04 |
| C5 | Language | -0.14 | -0.15 | -0.01 | -0.35 | -0.40 | -0.05 |

Note: EI = expected influence; BEI = bridge expected influence. “Original subgraph” refers to the 18‑symptom network obtained by removing the mRS node from the original 19‑node network (i.e., edges among symptoms were estimated in the presence of mRS). “Re‑estimated, no mRS” refers to a completely independent network estimation using the same 18 symptoms but excluding the mRS variable from the dataset. Δ = re‑estimated minus original subgraph. All values are based on Spearman correlations and EBIC glasso regularization (γ = 0.1). The Spearman rank correlation for EI across all 18 symptoms between the two networks was 1.00, and edge weights correlated at ρ = 0.993, confirming high stability.

**Supplementary Table 4.** Frequency distribution of HAMD item 3 (*Suicidality*) in the total cohort (n = 589)

| Score | Frequency (n) | Percentage (%) |
| --- | --- | --- |
| 0 | 530 | 90％ |
| 1 | 41 | 7％ |
| 2 | 14 | 2％ |
| 3 | 4 | 1％ |
| 4 | 0 | 0 |
| Total | 589 | 100％ |

**Supplementary Figure 1.** Flow chart of the study.

**Supplementary Figure 2.** Z-score standardized values of closeness, betweenness and strength for all nodes. Note: Closeness centrality refers to the inverse sum of the lengths of the shortest paths from a node to all other nodes. Betweenness centrality is the number of times a node lies on the shortest path between two other nodes. Strength centrality is the sum of all absolute values of edges connected to a node. Betweenness and closeness have demonstrated poor reliability in psychopathology network studies. Expected influence considers both positive and negative edges and may be more reliable and interpretable than strength.

**Supplementary Figure 3.** Z-score standardized values of bridge strength, bridge closeness, and bridge betweenness for all nodes.

**Supplementary Figure 4.** Accuracy of edge weights in the network Note: The red line depicts the sample edge weights, the black line represents bootstrap means, and the gray bar depicts the bootstrapped confidence interval.

**Supplementary Figure 5.** Bootstrap edge weights difference test between non-zero estimated edge-weights in the network. Note: Bootstrapped difference tests (α = 0.05) between edge-weights that were non-zero in the network. Significant differences between two edges are indicated by black boxes, non-significant differences are indicated by grey boxes. The color of the boxes (ranging from white to blue) corresponds to the thickness of the edge.

**Supplementary Figure 6.** Bootstrapped difference test for node expected influences in the network Note: Gray boxes indicate node expected influences that do not differ significantly from one another, while black boxes indicate node expected influences that do differ significantly.

**Supplementary Figure 7.** Bootstrapped difference test for node bridge expected influences in bridge network Note: Gray boxes indicate node bridge expected influences that do not differ significantly from one another, while black boxes indicate node bridge expected influences that do differ significantly.

**Supplementary Figure 8.** Stability of betweenness, closeness and strength in 1,000 case-dropping bootstraps. CS-C for betweenness: 0.52; CS-C for closeness: 0.52; CS-C for strength: 0.75.

**Supplementary Figure 9.** Stability of bridge betweenness, bridge closeness and bridge strength in 1,000 case-dropping bootstraps. CS-C for bridge betweenness: 0.36; CS-C for bridge closeness: 0.75; CS-C for bridge strength: 0.75.

**Supplementary Figure 10.** Comparison of network properties between participants older than 60 and those aged 60 or younger.

A Panel**:** A plot of bootstrap value of the maximum difference in any of the edge weights (1000 permutations). The difference was not significant (M=0.21, p=0.28)**.**

B Panel**:** A plot of bootstrap value of the difference in network global strength. The difference was not significant (network strength among above 60 participants: 6.63; among 60 and below 60: 5.28; p=0.73).

**Supplementary Figure 11.** Comparison of the 18‑symptom network derived from the original 19‑node network (left) versus the network re‑estimated after excluding the dysfunction node (right)

Note: D1 = Depressed mood, D2 = Feelings of guilt, D3 = Suicidality, D4 = Insomnia, D5 = Loss of interest, D6 = Retardation, D7 = Agitation, D8 = Psychiatric anxiety, D9 = Somatic anxiety, D10 = Gastrointestinal somatic, D11 = General somatic, D12 = Hypochondriasis, D13= Weight loss, C1 = Orientation, C2 = Registration, C3 = Attention, C4 = Memory, C5 = Language, DF= mRS score

Both networks contain the same 18 symptoms (13 HDRS items and 5 MMSE items). Edge colors represent positive (blue) or negative (red) partial correlations, and edge thickness is proportional to the absolute weight. The layout is identical between the two panels to facilitate visual comparison. The two networks are highly similar, with a Spearman correlation of edge weights of 0.993 and a median absolute edge‑weight difference of 0.0004, confirming that the core symptom structure is robust to the inclusion or exclusion of the dysfunction node.

**Supplementary Figure 12.** Lesion overlap map of the imaging subset (n = 429).

Overlap map showing the spatial distribution of ischemic lesions from 429 patients superimposed on the MNI152 template. The color bar indicates the absolute number of patients with a lesion at each voxel, with warmer colors representing greater overlap. Lesions were most frequently concentrated in the basal ganglia and thalamus, with involvement of adjacent subcortical white matter.
